# Prion Protein Modulates Cellular Iron Uptake: A Novel Function with Implications for Prion Disease Pathogenesis

**DOI:** 10.1371/journal.pone.0004468

**Published:** 2009-02-12

**Authors:** Ajay Singh, Maradumane L. Mohan, Alfred Orina Isaac, Xiu Luo, Jiri Petrak, Daniel Vyoral, Neena Singh

**Affiliations:** 1 The Department of Pathology, Case Western Reserve University, Cleveland, Ohio, United States of America; 2 Department of Pathological Physiology, First Faculty of Medicine, Charles University in Prague, Prague, Czech Republic; Swiss Federal Institute of Technology Lausanne, Switzerland

## Abstract

Converging evidence leaves little doubt that a change in the conformation of prion protein (PrP^C^) from a mainly α-helical to a β-sheet rich PrP-scrapie (PrP^Sc^) form is the main event responsible for prion disease associated neurotoxicity. However, neither the mechanism of toxicity by PrP^Sc^, nor the normal function of PrP^C^ is entirely clear. Recent reports suggest that imbalance of iron homeostasis is a common feature of prion infected cells and mouse models, implicating redox-iron in prion disease pathogenesis. In this report, we provide evidence that PrP^C^ mediates cellular iron uptake and transport, and mutant PrP forms alter cellular iron levels differentially. Using human neuroblastoma cells as models, we demonstrate that over-expression of PrP^C^ increases intra-cellular iron relative to non-transfected controls as indicated by an increase in total cellular iron, the cellular labile iron pool (LIP), and iron content of ferritin. As a result, the levels of iron uptake proteins transferrin (Tf) and transferrin receptor (TfR) are decreased, and expression of iron storage protein ferritin is increased. The positive effect of PrP^C^ on ferritin iron content is enhanced by stimulating PrP^C^ endocytosis, and reversed by cross-linking PrP^C^ on the plasma membrane. Expression of mutant PrP forms lacking the octapeptide-repeats, the membrane anchor, or carrying the pathogenic mutation PrP^102L^ decreases ferritin iron content significantly relative to PrP^C^ expressing cells, but the effect on cellular LIP and levels of Tf, TfR, and ferritin is complex, varying with the mutation. Neither PrP^C^ nor the mutant PrP forms influence the rate or amount of iron released into the medium, suggesting a functional role for PrP^C^ in cellular iron uptake and transport to ferritin, and dysfunction of PrP^C^ as a significant contributing factor of brain iron imbalance in prion disorders.

## Introduction

Prion protein (PrP^C^) is an evolutionarily conserved cell surface glycoprotein expressed abundantly on neuronal cells. Despite its ubiquitous presence, the physiological function of PrP^C^ has remained ambiguous. The best characterized role for this protein remains its involvement in the pathogenesis of familial, infectious, and sporadic prion disorders, where a change in the conformation of PrP^C^ from a mainly α-helical to a β-sheet rich PrP-scrapie (PrP^Sc^) form renders it infectious and pathogenic [1]–[5]. The mechanism by which PrP^Sc^ induces neurotoxicity, however, is not clear. Studies over the past decade have clarified several aspects of this process [1], [6], [7]. Prominent among these is the resistance of transgenic mice lacking neuronal PrP^C^ expression to PrP^Sc^ induced toxicity, implicating PrP^C^ as the principal mediator of the neurotoxic signal [8], [9]. However, prion infected transgenic mice expressing PrP^C^ only on astrocytes accumulate PrP^Sc^ and succumb to disease [10], leaving the matter unresolved. Adding to the complexity is the development of prion specific neuropathology in mice over-expressing normal or mutant PrP in the wrong cellular compartment in the absence of detectable PrP^Sc^, suggesting the presence of additional pathways of neurotoxicity [1], [7]. Although brain homogenates from these animals are not infectious in bioassays, these models suggest that a disproportionate change in the physiological function of PrP^C^ is as neurotoxic as the gain of toxic function by PrP^Sc^. Investigations on both fronts are therefore essential to uncover the underlying mechanism(s) of neurotoxicity in these disorders.

Efforts aimed at understanding the physiological function of PrP^C^ and pathological implications thereof have revealed several possibilities, varying with the model, the physiological state, and the extra- and intracellular milieu in a particular tissue. Some of the reported functions include a role in cell adhesion, signal transduction, and as an anti-oxidant and anti-apoptotic protein [7], [11], [12]. While the importance of these observations cannot be under-estimated, they fail to provide a direct link between PrP^C^ function and dysfunction to prion disease pathogenesis. In this context, it is interesting to note that PrP^C^ binds iron and copper, and is believed to play a functional role in neuronal iron and copper metabolism [13], [14]. Since both iron and copper are highly redox-active and neurotoxic if mis-managed, it is conceivable that dysfunction of PrP^C^ due to aggregation to the PrP^Sc^ form causes the reported accumulation of redox-active PrP^Sc^ complexes in prion infected cell and mouse models, inducing a state of iron imbalance [15]–[17]. A phenotype of iron deficiency in the presence of excess iron is noted in sporadic Cruetzfeldt-Jakob disease (sCJD) affected human and scrapie infected animal brain tissue, lending credence to this assumption [45].

To explore if PrP^C^ is involved in cellular iron metabolism, we investigated the influence of PrP^C^ and mutant PrP forms on cellular iron levels in human neuroblastoma cells expressing endogenous levels (M17) or transfected to express 6–7 fold higher levels of PrP^C^ or mutant PrP forms. The following parameters were evaluated: 1) total cellular iron, 2) intracellular labile iron pool (LIP), 3) iron content of ferritin, and 4) levels of iron uptake proteins transferrin receptor (TfR) and transferrin (Tf) and iron storage protein ferritin that respond to minor changes in the LIP [18], [19]. Our data demonstrate that PrP^C^ increases cellular iron levels and the cells demonstrate a state of mild overload, while pathogenic and non-pathogenic mutations of PrP alter cellular iron levels differentially, specific to the mutation.

## Results

### Normal and mutant PrP forms influence cellular iron levels differentially

**Figure 1:**
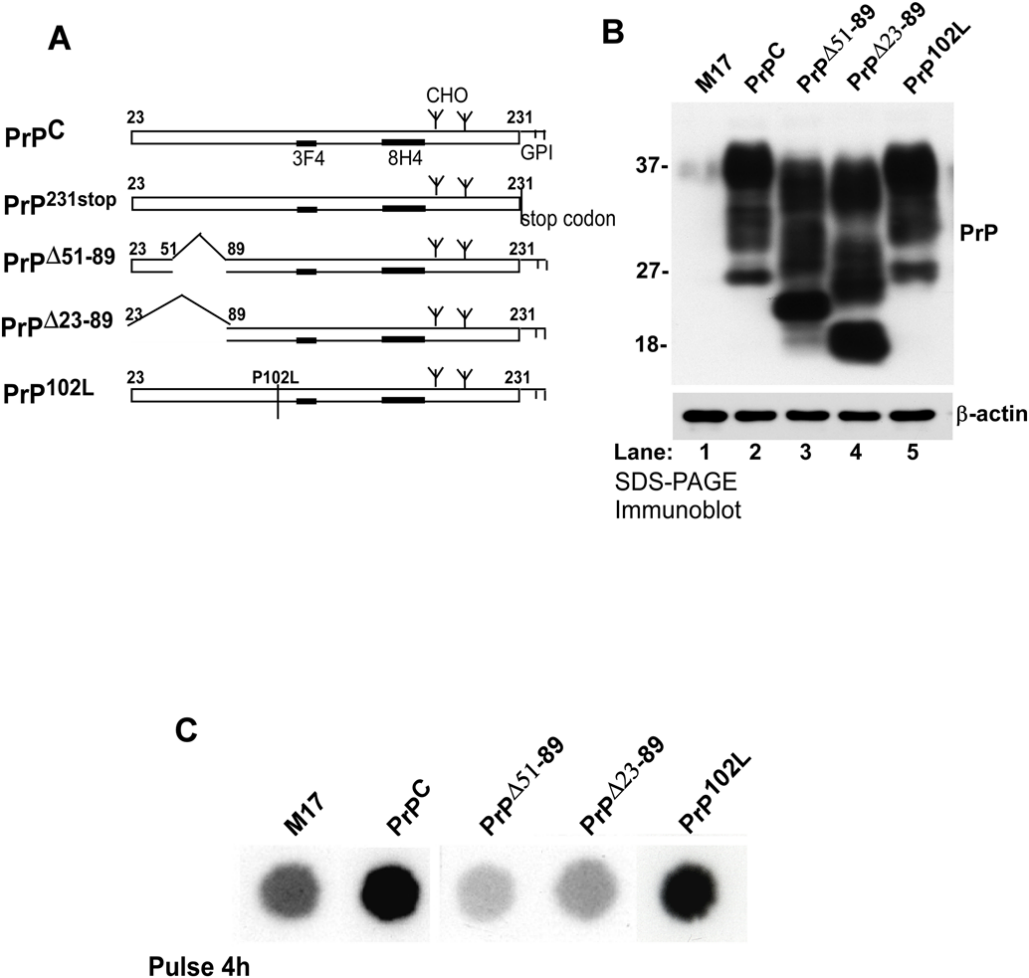
Cells expressing normal and mutant PrP forms incorporate different levels of iron. (A) Diagrammatic representation of PrP^C^ and mutant PrP forms evaluated in this study. (B) Lysates of M17, PrP^C^, PrP^Δ51–89^, PrP^Δ23–89^, and PrP^102L^ were resolved by SDS-PAGE and immunoreacted for PrP and β-actin. All transfected cell lines express 6–7 fold higher levels of PrP relative to non-transfected M17 cells (lanes 1–5). (C) Cell lines in (B) were radiolabeled with ^59^FeCl_3_-citrate complex, washed with PBS supplemented with 100 µM DFO to chelate surface bound iron, and lysed. Equal amount of protein from each sample was spotted on a PVDF membrane, air dried, and exposed to an X-ray film.

The influence of PrP expression on cellular iron status was evaluated in M17 cells expressing endogenous PrP^C^ or stably transfected to express 6–7 fold higher levels of PrP^C^ or the following mutant PrP forms: 1) PrP^231stop^ that lacks the glycosylphosphatidyl inositol (GPI) anchor and is secreted into the medium, 2) PrP^Δ51–89^ that lacks the copper binding octa-peptide repeat region, 3) PrP^Δ23–89^ that lacks the N-terminal 90 amino acids, and 4) PrP^102L^ associated with Gerstmann-Straussler-Scheinker disease (GSS), a familial prion disorder (Fig. 1A). Expression of PrP in transfected cell lines was assessed by separating cell lysates on SDS-PAGE and probing transferred proteins with the PrP specific monoclonal antibody 3F4 [26]. As expected, the di-, mono-, and unglycosylated forms of PrP^C^, PrP^Δ51–89^, PrP^Δ23–89^, and PrP^102L^ migrating between 20 and 37 kDa are detected (Fig. 1B, lanes 2–5). Deletion mutations PrP^Δ51–89^ and PrP^Δ23–89^ migrate faster than PrP^C^ and PrP^102L^ as expected (Fig. 1B, lanes 3 and 4). M17 lysates show barely detectable levels of PrP^C^, while transfected cell lines express significantly higher levels of PrP^C^ and mutant PrP forms (Fig. 1B, lanes 1–5).

To evaluate if PrP^C^ or mutant PrP forms influence cellular iron uptake, M17, PrP^C^, PrP^Δ51–89^, PrP^Δ23–89^, and PrP^102L^ cells cultured in serum-free medium for 1 hour were radiolabeled with ^59^FeCl_3_-citrate complex for 4 hours in the same medium, washed with PBS supplemented with 100 µM desferrioxamine (DFO) to remove surface bound iron, and lysed in non-denaturing buffer. Equal amount of protein from lysates was spotted on a PVDF membrane, air-dried, and exposed to an X-ray film. Surprisingly, PrP^C^ and PrP^102L^ cells incorporate significantly more ^59^Fe, while PrP^Δ51–89^, PrP^Δ23–89^ cells take up less ^59^Fe than M17 controls (Fig. 1C).

**Figure 2 pone-0004468-g002:**
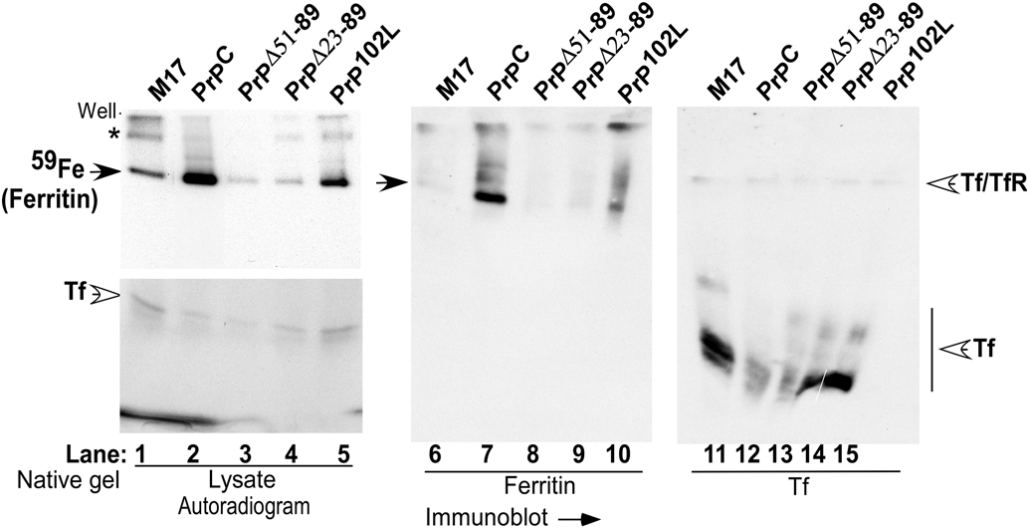
PrP influences iron incorporation in cellular ferritin. Radiolabeled lysates were fractionated on a 3–20% native gradient gel in duplicate. One set was subjected to autoradiography (lanes 1–5) and the other was transblotted and probed for ferritin and transferrin under native conditions (lanes 6–15).

Major ^59^Fe labeled proteins in these cells were identified by separating cell lysates prepared in non-denaturing buffer on a 3–20% native gel in duplicate. One part of the gel was dried and subjected to autoradiography (Fig. 2, lanes 1–5), while the other was transferred to a PVDF membrane under native conditions and probed for ferritin and Tf using specific antibodies [19], [20] (Fig. 2, lanes 6–15). Autoradiography shows a prominent iron labeled band consistent with ferritin (Fig. 2, lanes 1–5 and 6–10, black arrow), and a faster migrating band representing Tf (Fig. 2, lanes 1–5 and 11–15, open arrow) (the lower part of the autoradiograph is over-exposed to highlight the Tf band). Compared to M17 lysates, the amount of ^59^Fe bound to ferritin is higher in PrP^C^ and PrP^102L^ lysates, and lower in PrP^Δ51–89^ and PrP^Δ23–89^ lysates (Fig. 2, lanes 1–5). On the other hand, Tf bound iron is higher in M17 compared to PrP^C^, PrP^Δ51–89^, and PrP^Δ23–89^ lysates, and equivalent to PrP^102L^ lysates (Fig. 2, lanes 1–5 and 11–15). The slower migrating iron labeled bands (*) probably represent a complex of Tf and TfR (Fig. 2, lanes 1–5) [20], [21]. Probing for ferritin shows a major band and minor slower migrating forms probably representing ferritin complexes (Fig. 2, lanes 6–10, black arrow). Probing for Tf shows oligomers or glycosylation variants of Tf that correspond to ^59^Fe labeled purified transferrin fractionated similarly (Fig. 2, lanes 1–5, 11–15; Fig. S1). The relative levels of ferritin and Tf proteins in the samples correspond to radioactive iron in labeled ferritin and Tf bands in all samples (Fig. 2, lanes 1–15). Similar results were obtained when the cells were labeled with ^59^FeCl_3_-citrate complex for 16 hours or with purified ^59^Fe-Tf for 4 and 16 hours (data not shown), indicating similar uptake of non-transferrin and Tf bound Fe by these cells. Silver staining of re-hydrated autoradiographed gel confirms equal loading of protein for all samples analyzed (Fig. S1). Quantitative comparison of ferritin iron and levels of PrP, ferritin, and Tf between the cell lines is shown below in Fig. 4.

The identity of iron labeled bands in Fig. 2 was further confirmed by cutting each band from fractionated PrP^C^ lysates and re-fractionating electro-eluted proteins on SDS-PAGE followed by immunoblotting (Fig. S2). Lane 1 represents proteins eluted from the loading well that did not enter the running native gel. Lanes 2, 3 and 5 represent iron labeled bands that resolve adequately on native gels, and lane 4 represents unlabeled section of the gel that serves as a negative control. Sequential immunoreaction with specific antibodies confirms the presence of PrP in band 1, TfR in bands 1 and 2, ferritin in band 3, and Tf in band 5 (Fig. S2). Band 4 does not react with antibodies to known iron binding proteins. Silver staining shows co-migration of a few other un-identified proteins with bands 1–3, and almost none with bands 4 and 5 (Fig. S2).

**Figure 3:**
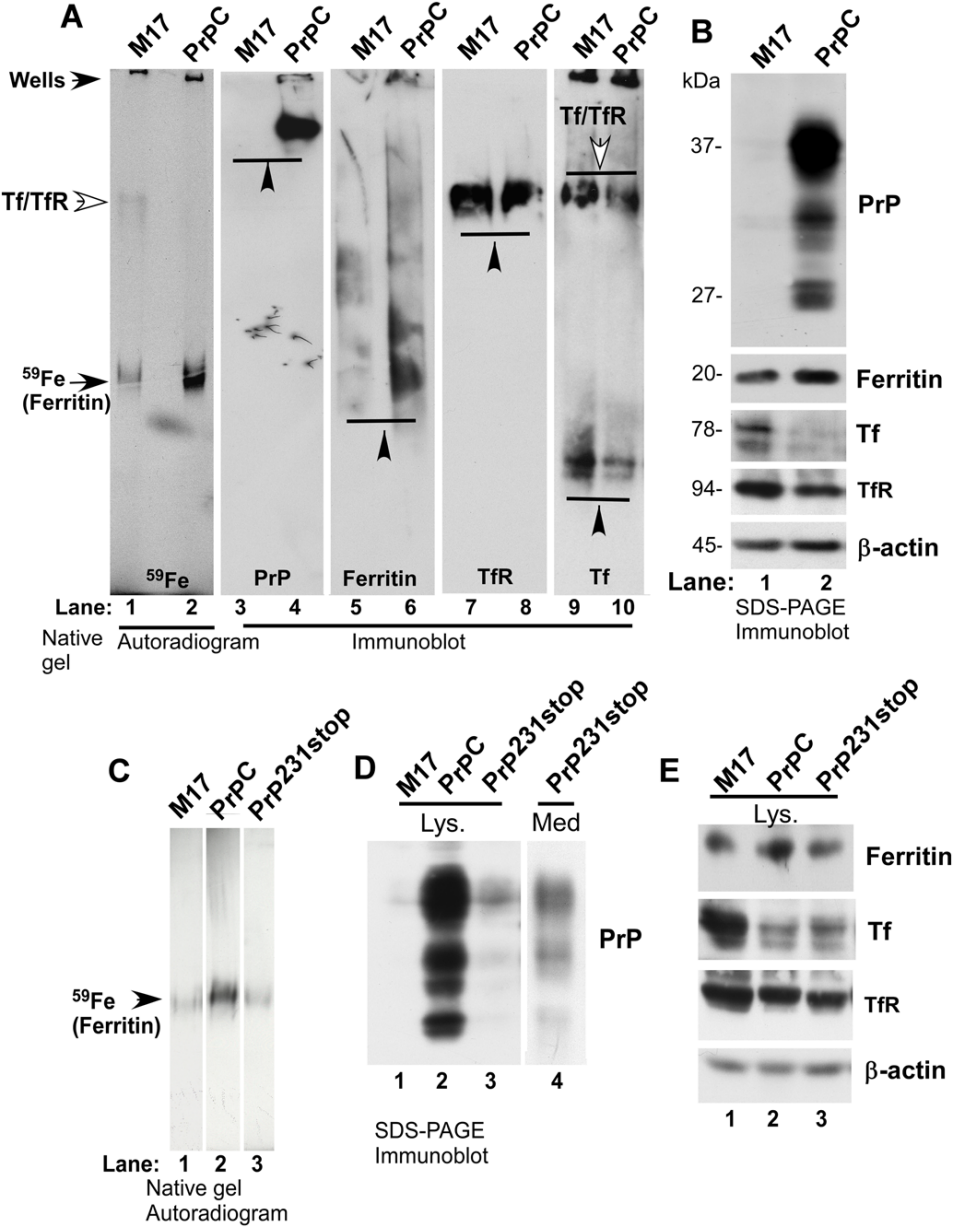
Expression of PrP on the plasma membrane is essential for iron incorporation in ferritin. (A) ^59^Fe-labeled M17 and PrP^C^ lysates were fractionated on a 3–9% native gradient gel and auto-radiographed (lanes 1 and 2), or immunoblotted as above with antibodies specific to PrP, ferritin, TfR, and Tf (lanes 3–10). (B) Immunoblotting of the same samples following fractionation by SDS-PAGE shows similar differences in the levels of PrP, ferritin, Tf, and TfR as in (A) after normalization with actin (lanes 1 and 2). (C) ^59^Fe-labeled M17, PrP^C^, and PrP^231stop^ lysates were fractionated by native gel electrophoresis and subjected to autoradiography (lanes 1–3). (D) Unlabeled lysates prepared from M17, PrP^C^, and PrP^231stop^ lysates, and methanol precipitated proteins from the medium sample of PrP^231stop^ cells were fractionated by SDS-PAGE and immunoblotted for PrP using 3F4 (lanes 1–4). (E) Membrane from (D) was re-probed for ferritin, Tf, TfR, and β-actin (lanes 1–3).

To determine if PrP^C^ mediates iron uptake directly, a modified non-denaturing gel system with a 3–9% gradient was used to separate ^59^Fe-labeled PrP effectively. Accordingly, M17 and PrP^C^ cells were radiolabeled with ^59^FeCl_3_-citrate complex for 4 hours as above, and lysates were fractionated in duplicate under non-denaturing conditions. One part was dried and exposed to an X-ray film, while the other was transferred to a PVDF membrane and probed for PrP, ferritin, TfR, and Tf. As in Fig. 2, the amount of ^59^Fe incorporated by ferritin in PrP^C^ cells is significantly higher than M17 cells (Fig. 3A, lanes 1 and 2, black arrow). A slower migrating ^59^Fe labeled band corresponding to Tf/TfR complex is detected in M17 lysates (Fig. 3A, lanes 1, 7, and 9, open arrow). Unlike Figure 2, PrP is resolved on this less concentrated gel system and is detected by PrP specific antibody 3F4 (Fig. 3A, lane 4, arrow-head). However, a corresponding ^59^Fe labeled band is not detected in lane 2, though pure ^59^Fe-labeled recombinant PrP is readily detected by this method as demonstrated previously [17]. Evaluation of iron modulating proteins shows higher levels of ferritin and lower levels of TfR and Tf in PrP^C^ lysates relative to M17 as in Fig. 1 above (Fig. 3A, lanes 5–10, arrow-head). Ferritin and Tf/TfR complex show corresponding iron labeled bands as expected (Fig. 3A, compare lanes 1, 2 with 5, 6, 9, 10). Fractionation of the same samples by SDS-PAGE followed by immunoblotting confirms increased levels of ferritin and decreased levels of Tf and TfR in PrP^C^ lysates compared to M17 controls (Fig. 3B, lanes 1 and 2). Together, these results demonstrate that PrP^C^ increases total cellular iron, ferritin iron, and ferritin levels, and decreases Tf and TfR levels. However, the absence of ^59^Fe-labeled PrP^C^ indicates that either the association of PrP with ^59^Fe is transient or relatively weak and disrupted after cell lysis, or alternatively, PrP facilitates the incorporation of ^59^Fe into ferritin by an indirect mechanism that does not involve the formation of a PrP-iron complex.

To evaluate if expression of PrP^C^ on the cell surface is required for iron uptake, a similar evaluation was carried out in cells expressing PrP^231stop^ that lacks the GPI anchor and is secreted into the medium. Radiolabeling of M17, PrP^C^, and PrP^231stop^ cells with ^59^FeCl_3_-citrate complex for 4 hours shows significantly more ^59^Fe-ferritin in PrP^C^ cells compared to M17 as above, and minimal change in PrP^231stop^ samples (Fig. 3C, lanes 1–3, black arrow). Western blotting of M17, PrP^C^, and PrP^231stop^ lysates and medium sample from PrP^231stop^ cells cultured overnight in serum-free medium with 3F4 shows the expected glycoforms of PrP in PrP^C^ lysates, and undetectable reactivity in M17 and PrP^231stop^ lysates as expected (Fig. 3D, lanes 1–3). However, significant reactivity is detected in the medium of PrP^231stop^ cells, demonstrating adequate expression and secretion of PrP^231stop^ in transfected cells (Fig. 3D, lane 4) [22], [23]. Re-probing of lysate samples for ferritin, Tf, and TfR shows increased levels of ferritin and decreased levels of Tf and TfR in PrP^C^ samples compared to M17 lysates (Fig. 3E, lanes 1 and 2). PrP^231stop^ lysates show minimal change in ferritin levels, and surprisingly, lower levels of Tf and TfR relative to M17 lysates (Fig. 3E, lanes 1 and 3). This observation is surprising since ^59^Fe-ferritin levels in PrP^231stop^ cells are as low as M17, and yet the cells do not show increased levels of Tf and TfR as in M17-cells. Reaction for β-actin confirms equal loading of protein in all samples (Fig. 3E, lanes 1–3).

**Figure 4:**
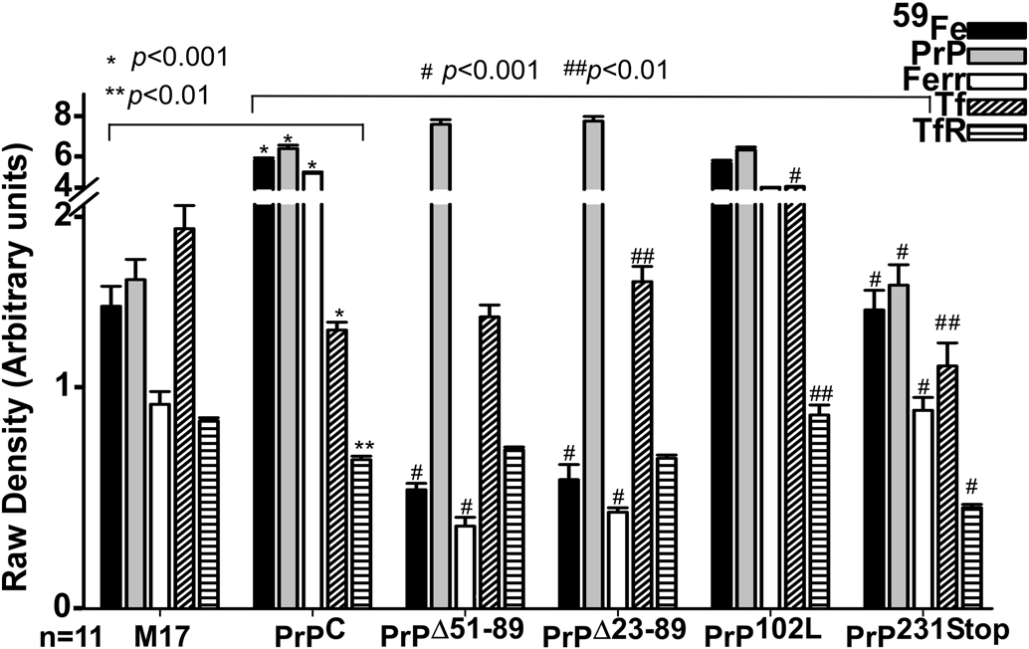
Quantitative analysis of the results in Figures 1–3. Quantitative evaluation after densitometry of ferritin iron and levels of PrP, ferritin, Tf, and TfR in PrP^C^, PrP^Δ51–89^, PrP^Δ23–89^, PrP^102L^, PrP^231stop^-cells relative to non-transfected M17 controls. Values are mean±SEM of 11 independent experiments. The y-scale is linear but has been re-scaled after the break to illustrate the data clearly. For M17 *vs.* PrP^C^ *p<0.001, **p<0.01, and for PrP^C^ *vs.* mutant cell lines ^#^ *p*<0.001, ^##^ *p*<0.01).

Quantitative comparison of ferritin iron and levels of ferritin, Tf, and TfR shows significant differences between cell lines. Thus, relative to M17 cells, PrP^C^ cells show an increase in ferritin iron and ferritin levels to 570 and 565%, and a decrease in Tf and TfR levels to 70 and 75% respectively. A similar comparison of mutant cell lines relative to PrP^C^ cells shows the following: PrP^Δ51–89^ cells show a decrease in ferritin iron and ferritin to 7.0, 6.9%, and insignificant change in Tf and TfR levels. PrP^Δ23–89^ cells show a similar decrease in ferritin iron and ferritin levels to 7.5 and 7.2%, an increase in Tf to 120%, and insignificant change in TfR levels. PrP^102L^-cells show a decrease in ferritin iron and ferritin levels to 89 and 90%, and an increase in Tf and TfR levels to 300 and 142% respectively. PrP^231stop^ cells show a decrease in ferritin iron and ferritin to 27 and 16%, and a decrease in Tf and TfR levels to 89 and 67% respectively. Quantification of PrP expression relative to M17 shows levels of 650, 710, 750, 610, and 5% in PrP^C^, PrP^Δ51–89^, PrP^Δ23–89^, PrP^102L^, and PrP^231stop^ cells respectively (Fig. 4).

Considering the tightly orchestrated and coordinated balance between cellular iron levels and iron uptake and storage proteins [18], [21], these results indicate a mild iron overload in PrP^C^-cells relative to M17-cells, and an indefinable phenotype in mutant cell lines since the iron uptake proteins Tf and TfR do not respond to ferritin iron levels as expected. Since Tf and TfR levels are reflective of the biologically available intracellular labile iron pool (LIP) that is maintained within the physiological range by ferritin, these results indicate a disconnect between ferritin iron and the cellular LIP, or a failure of the iron regulatory loop involving the LIP, iron binding proteins 1 and 2, TfR, and ferritin to induce appropriate response.

### Mutant PrP forms influence the uptake of iron by ferritin

**Figure 5:**
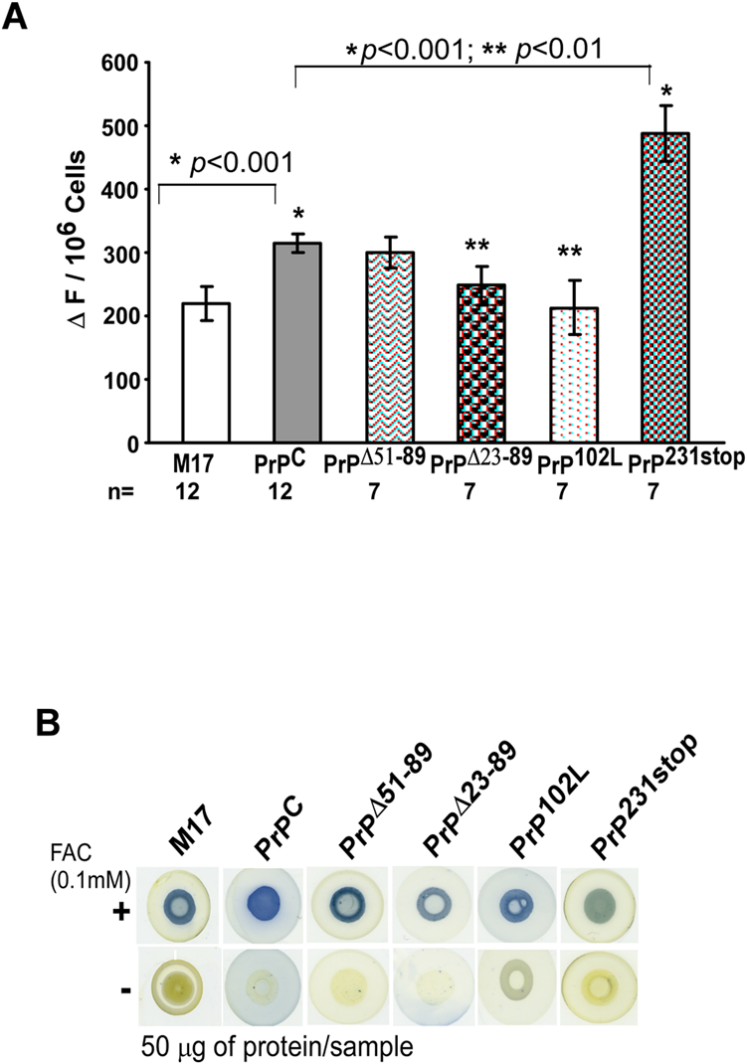
Cells expressing normal and mutant PrP forms show differential levels of LIP and uptake of extra-cellular iron. (A) Indicated cell lines were loaded with calcein and intracellular LIP was estimated by quantifying the SIH chelatable iron pool. Values are mean±SEM. n = 12 for M17 and PrP^C^, and 7 for mutant cell lines. *p<0.001, **p<0.01, ^#^ *p*<0.001, ^##^ *p*<0.01. (B) The same cell lines were exposed to 0.1 mM FAC for 16 hours and 50 µg of protein from cell homogenates was spotted on a PVDF membrane and reacted with Ferene-S, a dye that forms a blue reaction product with iron [25].

The influence of normal and mutant PrP forms on intracellular LIP was evaluated in M17 and transfected cell lines cultured in complete medium under normal culture conditions. All cell lines were loaded with the iron binding dye calcein-AM, and the increase in fluorescence in response to salicylaldehyde isonicotinoyhydrazone (SIH), a cell permeable iron chelator, was measured (Fig. 5A) [24]. Relative to M17 cells, PrP^C^ cells show an increase in LIP to 143%, an expected observation since the ferritin iron levels of these cells are also higher than M17 cells (compare Figs. 5A and 4). A similar evaluation of mutant cell lines relative to PrP^C^-cells shows a decrease in LIP to 95, 78 and 67% in PrP^Δ51–89^, PrP^Δ23–89^, PrP^102L^-cells, and an increase to 155% in PrP^231stop^ cells respectively (Fig. 5A). These results indicate that Tf and TfR levels in mutant cell lines observed in Fig. 4 above respond to the LIP rather than ferritin iron content as expected. More importantly, these results indicate a block in uptake or increased uptake of iron by ferritin in specific cell lines, accounting for the disproportionate levels of ferritin iron and intracellular LIP, and the unexpected response of Tf and TfR to cellular iron content.

To evaluate if the difference in ferritin iron content of different cell lines is maintained in the presence of excess extra-cellular iron, M17, PrP^C^, PrP^Δ51–89^, PrP^Δ23–89^, PrP^102L^, and PrP^231stop^-cells were cultured overnight in the presence of 0.1 mM ferric ammonium citrate (FAC). (This dose of FAC was found to cause <1% cell death after overnight exposure). After washing the cells with PBS supplemented with 100 µM DFO to remove surface bound iron, cells were disrupted with glacial acetic acid and equal amount of protein from each cell line was spotted on a PVDF membrane. Reaction with Ferene-S, a dye that forms a blue reaction product with iron [25], shows a marked increase in protein bound iron in all cell lines compared to unexposed controls (Fig. 5B). More importantly, each cell line reflects cell-specific differences in protein bound iron as observed for ferritin iron above (Fig. 5B). Fractionation of lysates by SDS-PAGE followed by immunoblotting for PrP, ferritin, and TfR shows up-regulation of PrP and ferritin, and down-regulation of TfR to undetectable levels in FAC exposed lysates (Fig. S3 A, lanes 1–4) [17]. Up-regulation of PrP in response to FAC appears to be at the mRNA level (Fig. S3 B). These results suggest a dominant role for PrP in the transport of extracellular iron to ferritin both under normal culture conditions and in the presence of excess extra-cellular iron.

Together, the above results demonstrate a state of relative iron overload in PrP^C^-cells compared to M17 controls as indicated by an increase in intracellular LIP and iron content of ferritin, increase in iron storage protein ferritin, and decrease in iron uptake proteins Tf and TfR. Relative to PrP^C^-cells, mutant PrP expressing cells show a substantial decrease in ferritin iron in PrP^Δ51–89^, PrP^Δ23–89^, and PrP^231stop^-cells, and relatively less reduction in PrP^102L^-cells. Intracellular LIP is reduced in PrP^Δ23–89^ and PrP^102L^, minimally altered in PrP^Δ51–89^, and substantially increased in PrP^231stop^-cells relative to PrP^C^-cells. Tf and TfR respond to LIP levels in some cell lines, but show an unexpected change in others, reflecting a state of cellular iron imbalance.

### Stimulation of PrP endocytosis increases, and cross-linking decreases ferritin iron content

Further support for the role of PrP in mediating cellular iron uptake was obtained by assessing iron incorporation into ferritin following stimulation or disruption of PrP^C^ endocytosis by 3F4, a well characterized monoclonal antibody specific for methionine residues 109 and 112 of human PrP [26]. A similar approach has been used successfully to down-regulate mouse PrP using Fab fragments of PrP specific antibodies [27]. Initial evaluation revealed that 3F4 concentrations of 1 and 12 µg/ml are optimal for stimulating and disrupting endocytosis of PrP^C^ respectively without compromising cell viability.

**Figure 6:**
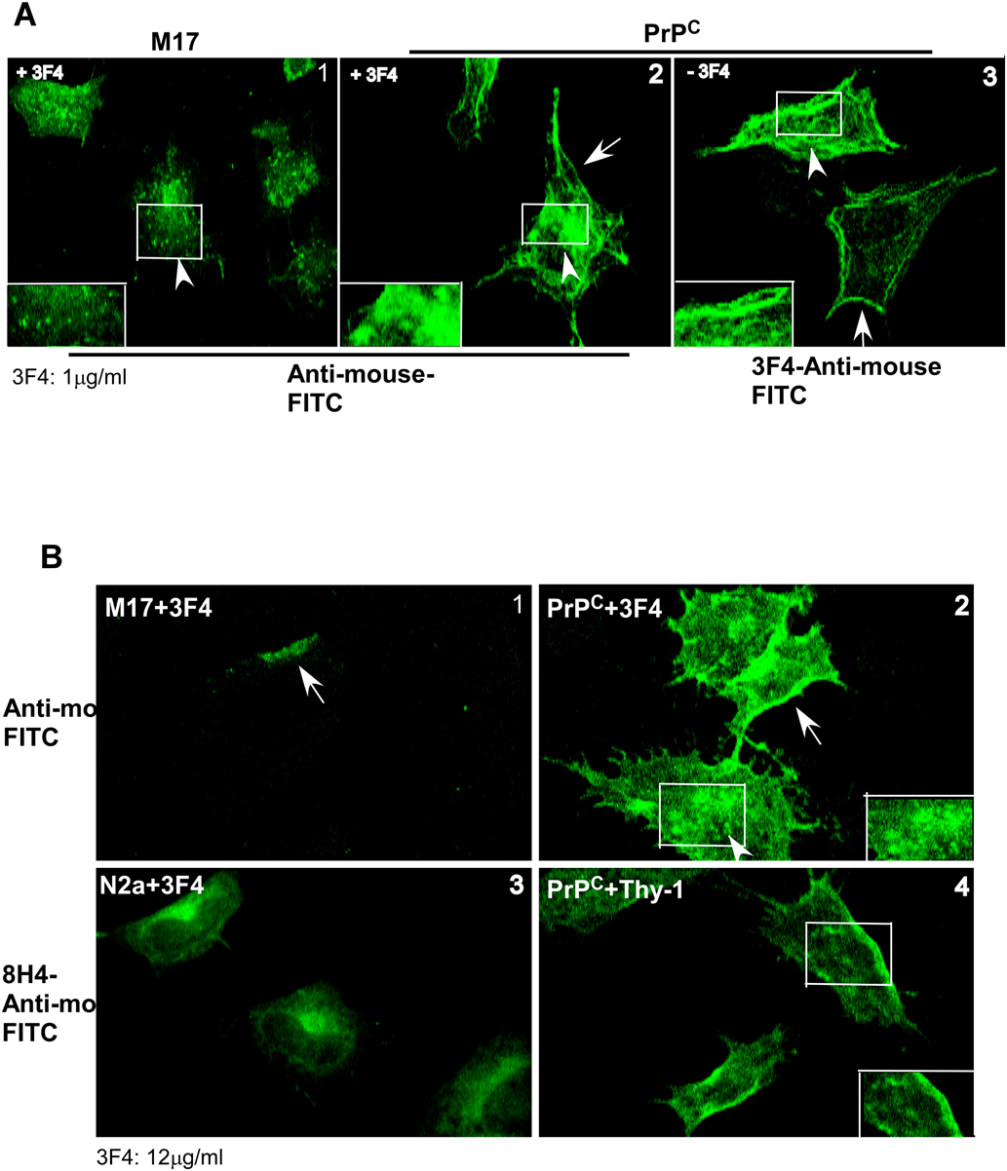
Exposure of PrP^C^-cells to different concentrations of 3F4 induces endocytosis or cross-linking of PrP. (A) Immunostaining of M17 and PrP^C^ cells exposed to 1 µg/ml of 3F4 for 5 days shows a prominent reaction in vesicular structures in M17 and PrP^C^ cells (panels 1 and 2). Coalesced vesicles simulating aggregated PrP^C^ are evident near the Golgi region and in the cytosol of PrP^C^ cells (panel 2). Untreated PrP^C^-cells reacted with 8H4-anti-mouse-FITC show a prominent reaction at the plasma membrane as expected (panel 3). (B) Reaction of M17 and PrP^C^ cells exposed to 12 µg/ml of 3F4 for 4 hours with anti-mouse FITC shows cross-linking of PrP on the plasma membrane of M17 and PrP^C^ cells (panels 1 and 2, arrow) and a slight increase of reactivity in vesicular structures in the latter (panel 2, arrow-head). Similar exposure of N2a-cells to 3F4 and PrP^C^-cells to anti-Thy1 antibody followed by immunoreaction with 8H4-anti-mouse-FITC shows plasma membrane and Golgi reaction of endogenous PrP in N2a cells (panel 3) and plasma membrane distribution of PrP in Thy-1 exposed cells (panel 4). (Mouse PrP expressed by N2a cells does not react with 3F4).

To evaluate the effect of antibody treatment morphologically, M17 and PrP^C^-cells exposed to 1 µg/ml of 3F4 for 5 days were fixed, permeabilized, and reacted with anti-mouse-FITC. Both M17 and PrP^C^-cells show minimal reactivity at the plasma membrane, but significant reactivity in endocytic vesicles that are more prominent in PrP^C^ cells (Fig. 6A, panels 1 and 2, arrow-head). These observations suggest significant endocytosis of PrP^C^ along with 3F4. Untreated PrP^C^-cells reacted with 3F4-anti-mouse-FITC show punctuate reaction at the plasma membrane and minimal intracellular reaction as expected for normal distribution of PrP^C^ (Fig. 6A, panel 3, arrow). Exposure to 12 µg/ml of 3F4, however, cross-links PrP^C^ at the plasma membrane and reduces its endocytosis significantly (Fig. 6B, panels 1 and 2). As a control, mouse neuroblastoma cells (N2a) expressing mouse PrP that does not react with 3F4 were exposed to 3F4 and reacted with mouse PrP-specific antibody 8H4 followed by anti-mouse-FITC. Examination shows normal distribution of PrP^C^ at the plasma membrane and some reactivity in the Golgi region as expected (Fig. 6B, panel 3) [26]. Exposure of PrP^C^ cells to anti-Thy-1, a monoclonal antibody to an irrelevant GPI-linked protein abundant on neuronal cells shows normal distribution of PrP^C^ when reacted with 8H4-anti-mouse-FITC (Fig. 6B, panel 4), confirming the specificity of 3F4 mediated endocytosis and cross-linking of PrP^C^.

**Figure 7:**
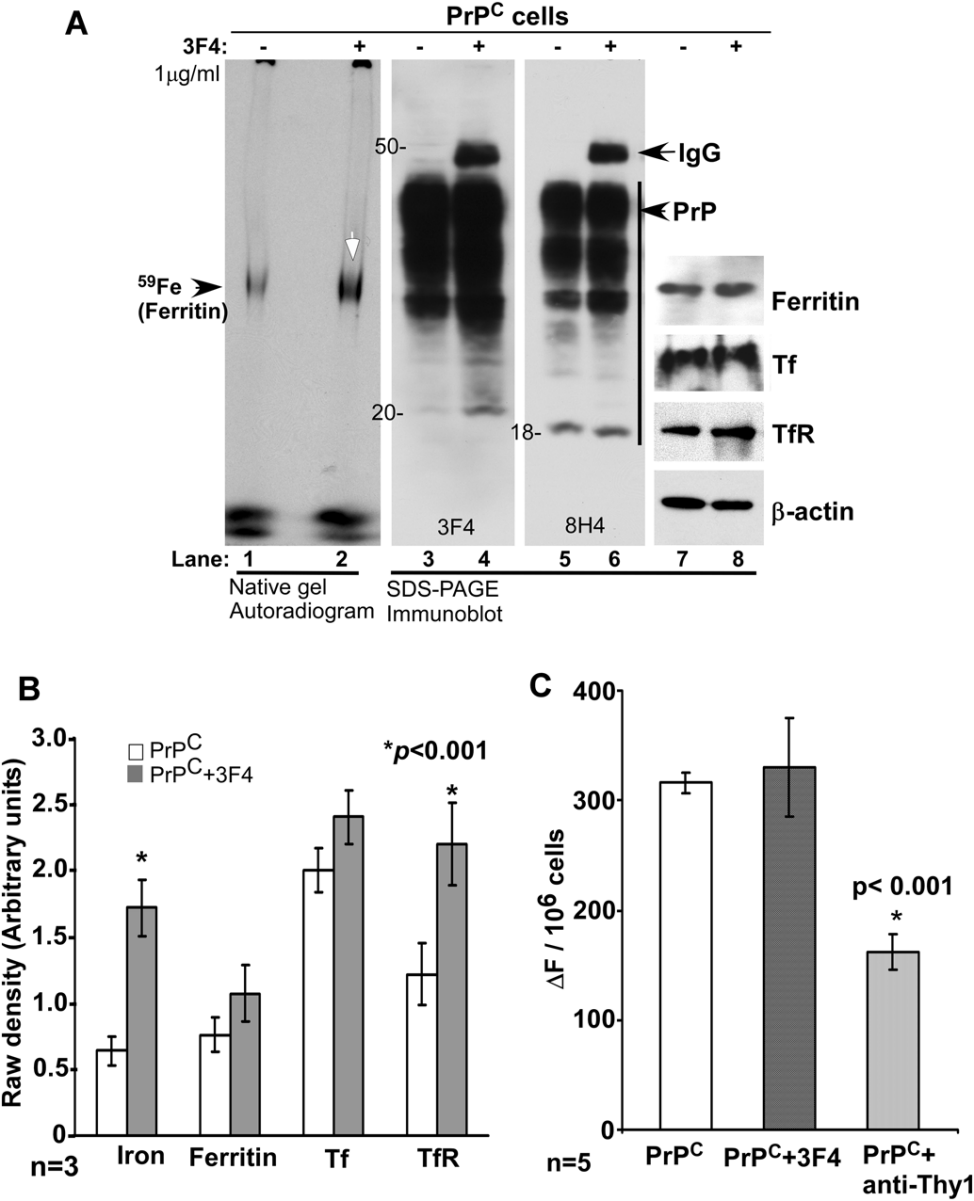
Endocytosis of PrP increases ferritin iron content. (A) PrP^C^-cells exposed to 1 µg/ml of 3F4 for 5 days were radiolabeled with ^59^FeCl_3_ for 4 hours, and lysates were fractionated on a non-denaturing gel and auto-radiographed (lanes 1 and 2). Equal aliquots of the same samples were boiled in SDS-containing sample buffer and fractionated in duplicate by SDS-PAGE followed by immunoblotting with PrP specific antibodies 3F4 and 8H4 (lanes 3–6). Subsequently, the membranes were re-probed for ferritin, Tf, TfR, and β-actin (lanes 7 and 8). (B) Quantification by densitometry shows an increase in ferritin iron and TfR levels, and insignificant change in Tf levels in 3F4 exposed cells. Values are mean±SEM of three independent experiments. *p<0.001 compared to untreated cells. (C) Estimation of LIP after exposing the cells to 1 µg/ml of 3F4 or anti-Thy-1 antibody for 5 days shows insignificant difference between untreated and 3F4 treated PrP^C^ cells, and a decrease in anti-Thy-1 treated cells. *p<0.001. n = 5.

The effect of increased endocytosis of PrP^C^ on ferritin iron content was evaluated by radiolabeling cells cultured in the presence of 1 µg/ml of 3F4 with ^59^FeCl_3_ for the last 4 hours of the incubation, and analyzing radiolabeled lysates as in Figure 1 above. Fractionation by non-denaturing page shows a significant increase in ferritin iron in the 3F4 exposed lysate compared to untreated control (Fig. 7A, lanes 1 and 2, open arrow). Analysis by SDS-PAGE and immunoblotting shows 2–3 fold increase in reactivity for all PrP glycoforms with anti-PrP antibodies 3F4 and 8H4 (Fig. 7A, lanes 3–6). However, the 18 kDa fragment that results from recycling of PrP^C^ from the plasma membrane is not increased in 3F4 exposed lysates, indicating stimulation of PrP^C^ internalization and possible intracellular accumulation by 3F4 binding rather than increased recycling from the plasma membrane (Fig. 7A, lanes 5 and 6) [28]. The 50 kDa band represents internalized 3F4 (Fig. 7A, lanes 4 and 6). Immunobloting for ferritin, Tf, and TfR shows an increase in TfR, and minimal change in ferritin and Tf levels (Fig. 7A, lanes 7 and 8). Quantification by densitometry shows an increase in ferritin iron to 271%, and insignificant change in ferritin and Tf levels by 3F4 treatment. The increase in TfR levels to 175% is probably due to co-endocytosis with PrP-antibody complex (Fig. 7B). Measurement of cellular LIP revealed insignificant difference between 3F4 exposed and untreated controls after 24 hours (data not shown) or 5 days of treatment, indicating efficient transport of iron to ferritin within this time frame (Fig. 7C). PrP^C^ cells treated with anti-Thy-1 antibody, however, demonstrated a significant decrease in LIP after 5 days of incubation with 3F4 (Fig. 7C).

**Figure 8:**
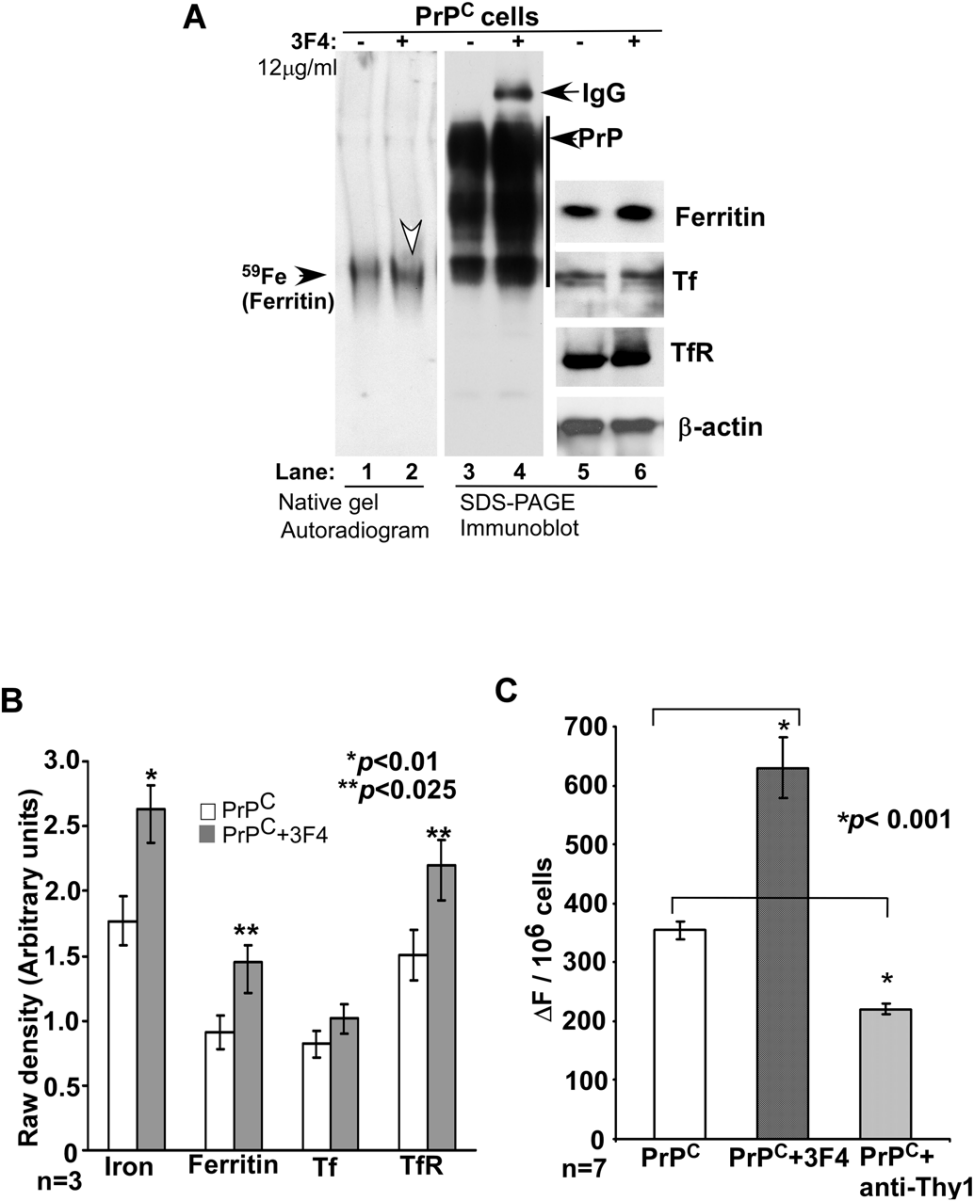
Cross-linking of PrP has minimal effect on ferritin iron content. (A) PrP^C^-cells exposed to 12 µg/ml of 3F4 for 4 hours were radiolabeled with ^59^FeCl_3_ in the last 2 hours, and lysates were fractionated on a native gel followed by autoradiography (lanes 1 and 2). Equal aliquots of lysates were fractionated by SDS-PAGE as above and immunoblotted with 3F4 (lanes 3 and 4). The membrane was re-pobed for ferritin, Tf, TfR, and β-actin (lanes 5 and 6). (B) Quantification by densitometry shows an increase in ferritin iron, ferritin, and TfR levels, and insignificant change in Tf levels by 3F4 treatment. *p<0.001, **p<0.025. n = 3. (C) Estimation of LIP after exposing the cells to 12 µg/ml of 3F4 or anti-Thy-1 antibody for 4 hours shows an increase in 3F4 exposed cells, and a decrease in anti-Thy-1 treated cells. *p<0.001. n = 7.

A similar evaluation of cells exposed to 12 µg/ml of 3F4 for 4 hours shows significantly less increase in ferritin iron compared to untreated controls (Fig. 8A, lanes 1 and 2, open arrow). Separation by SDS-PAGE and immunoblotting shows increase in PrP reactivity (Fig. 8A, lane 4) and an increase in the levels of ferritin, Tf, and TfR (Fig. 8A, lanes 5 and 6). Quantification shows an increase in ferritin iron to 148%, and an increase in the levels of ferritin and TfR to 153 and 146% respectively. Tf levels show insignificant change by this treatment (Fig. 8B). A similar increase in ferritin iron is observed when M17 cells expressing endogenous levels of PrP are exposed to 3F4 (Fig. S4 lanes 1 and 2), ruling out the effect of over-expression of PrP^C^ on these observations. Exposure to equivalent amounts of anti-Thy-1 does not alter ferritin iron content significantly (Fig. S4, lane 3). Measurement of intracellular LIP after 4 hours of exposure to 12 µg/ml of 3F4 shows an increase to 170% in treated cells compared to untreated controls. Exposure to similar concentrations of anti-Thy-1 shows a decrease to 70% (Fig. 8C), an unexpected effect that requires further evaluation.

The above results indicate that stimulation of PrP^C^ endocytosis over a prolonged period increases iron incorporation into ferritin, whereas cross-linking of PrP^C^ that is likely to result in its degradation following endocytosis has relatively less effect on ferritin iron. The increase in intra-cellular LIP by cross-linking PrP without any increase in ferritin iron probably reflects inefficient transport of iron to ferritin in the absence of PrP, as observed for certain mutant forms of PrP. The levels of ferritin, Tf, and TfR probably reflect an artifactual change due to membrane perturbation by antibody treatment rather than a response to intracellular LIP.

### PrP does not modulate release of iron from cells

**Figure 9:**
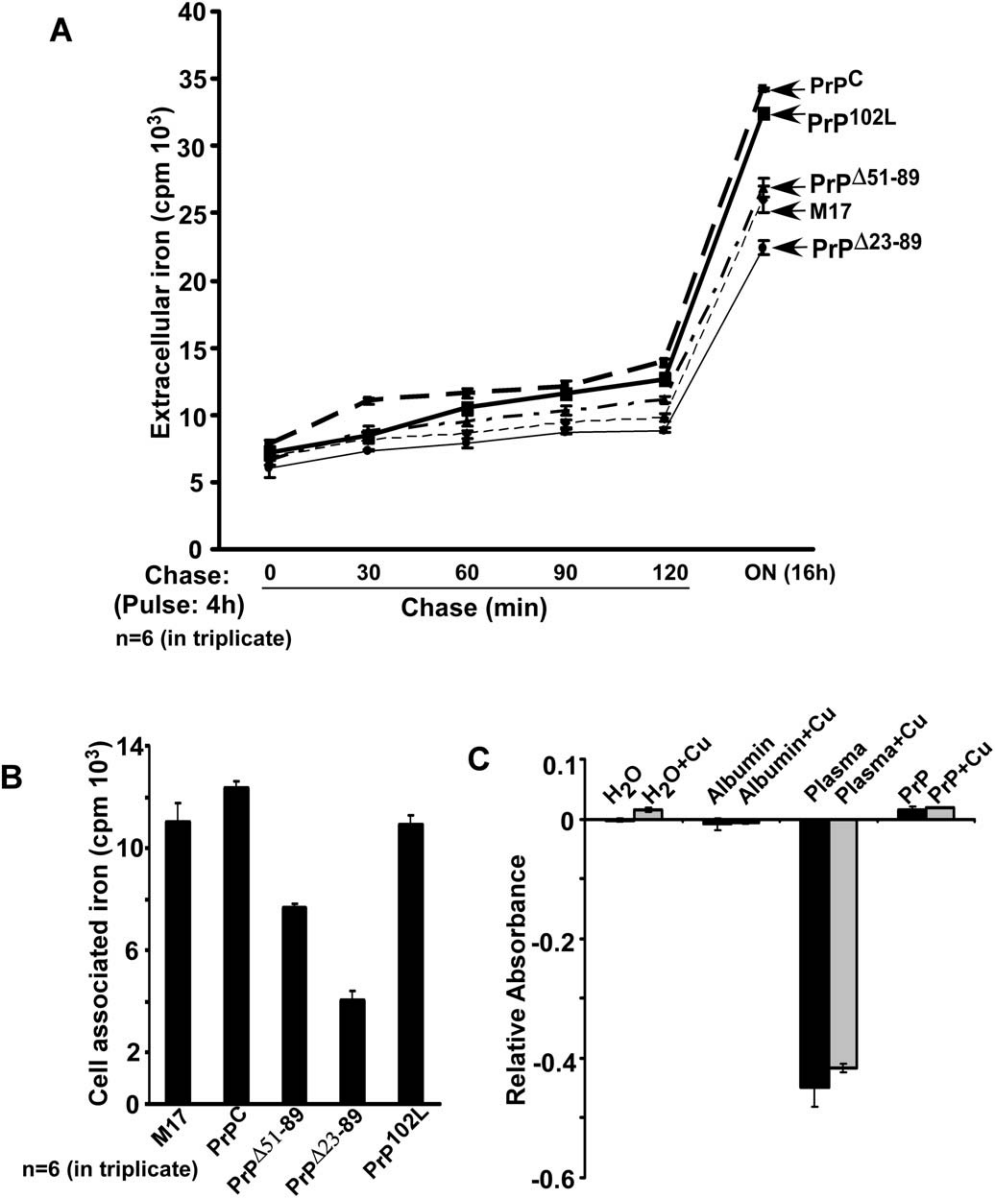
PrP is not involved in the export of iron from cells. (A) Cells expressing PrP^C^, PrP^Δ51–89^, PrP^Δ23–89^, and PrP^102L^ were radiolabeled with ^59^FeCl_3_, washed with PBS supplemented with DFO, and chased in complete medium for 30, 60, 90, 120 min, and 16 hours. At the indicated time points equal aliquots of medium samples were quantified in a γ-counter. Estimation of released ^59^Fe does not show a significant difference between the indicated cell lines at any time point. n = 6 experiments in triplicate. (B) Cell associated ^59^Fe after 16 h of chase reflects the ferritin iron content of each cell line noted in Figure 1 above, though the difference between cell lines is significantly less. (C) Possible ferroxidase activity of recombinant PrP was measured using the established colorimetric method [44] with modifications. Negative controls included water and albumin supplemented with copper, and positive controls included plasma in the absence or presence of copper. Recombinant PrP does not show detectable ferroxidase activity either in the absence or presence of copper, whereas plasma shows a robust reaction under similar conditions.

To determine if the difference in cellular iron levels between cell lines is due to differential release into the medium, M17, PrP^C^, PrP^Δ51–89^, PrP^Δ23–89^, and PrP^102L^ cells were cultured in the presence of ^3^H-thymidine overnight to monitor cell proliferation and radiolabeled with ^59^FeCl_3_ for 4 hours as above. Labeled cells were washed with PBS containing 100 µM DFO to remove surface bound ^59^Fe, and chased in complete medium for 30 minutes to 16 hours. At the indicated times equal aliquots of medium were retrieved and released ^59^Fe was quantified in a γ-counter. Kinetic analysis shows minimal difference in extracellular iron between cell lines after normalizing with ^3^H-thymidine (Fig. 9A). Estimation of cell-associated ^59^Fe after 16 hours of chase shows more ^59^Fe in PrP^C^ and PrP^102L^, and significantly less in PrP^Δ51–89^ and PrP^Δ23–89^ compared to M17 lysates as observed in Fig. 1 above (Fig. 9B). However, the fold difference in ferritin iron content between M17 and other cell lines is significantly less after 16 hours of chase, and represents steady state levels of iron content in each cell line. Evaluation of possible ferroxidase activity of recombinant PrP using plasma as a positive control yielded negative results (Fig. 9C). Though informative, this result does not rule out possible ferroxidase activity of cell-associated PrP, a technically challenging assay that has yielded inconclusive results (data not shown).

## Discussion

The results presented in this report demonstrate an unprecedented role of PrP in facilitating iron uptake by cells and its transport to cellular ferritin. Using a combination of neuroblastoma cell lines expressing normal and mutant PrP forms, we demonstrate that over-expression of PrP^C^ increases intracellular LIP and the amount of iron deposited in ferritin. Pathogenic and non-pathogenic mutations of PrP over-expressed to the same extent as PrP^C^ alter cellular LIP and ferritin iron content differentially, specific to the mutation. Certain cell lines, especially cells expressing anchorless PrP^231stop^, demonstrate increased LIP in the presence of decreased ferritin iron, while PrP^102L^-cells display low LIP in the presence of adequate ferritin iron. Furthermore, stimulation of endocytosis by PrP specific antibody increases ferritin iron, while cross-linking at the plasma membrane increases LIP but has minimal effect on ferritin iron, indicating that alteration of PrP function or cellular localization disturbs the homeostasis between ferritin iron and cellular LIP. The differential incorporation of iron by mutant cell lines is maintained in the presence of excess extra-cellular iron, demonstrating a dominant role of PrP^C^ in iron uptake and transport. The positive effect of PrP^C^ on cellular iron is mainly due to enhanced uptake since the amount released into the culture medium is not altered in any of the cell lines tested. Together, these observations suggest a role for PrP^C^ in mediating iron uptake and transport to ferritin directly, or by interacting with other iron modulating proteins. Below we discuss these data with reference to possible functions of PrP^C^ in cellular iron metabolism, and the implications thereof in inducing imbalance in iron homeostasis observed in prion disease affected brains [15], [16], [45].

It is surprising that a GPI-linked protein such as PrP^C^ is involved in iron transport to ferritin since PrP^C^ is a membrane protein that undergoes vesicular transport while ferritin is cytosolic [29]. Normally, cellular iron uptake is mediated by the Tf/TfR dependent and independent pathways, the former being most prominent and well characterized especially in neuroblastoma cells. In the Tf/TfR dependent pathway, ferric iron captured by Tf is taken up by the cells through TfR-mediated uptake *via* clathrin coated pits. Tf-bound ferric iron is released in the acidic environment of the endosomes, reduced to ferrous iron by an endosomal ferric reductase Steap3, and transported across the endosomal membrane by DMT1 to cytosolic ferritin where it is oxidized to the fairly inert ferric form by ferritin H-chain and stored [18], [19], [29]. In the Tf-independent pathway, iron is taken up by an unknown transport mechanism, possibly non-specifically by fluid phase of endocytosis, and stored in ferritin. Ferritin regulates the biologically available LIP in the cell, and is itself regulated by iron regulatory proteins (IRPs) 1 and 2 [18], [19], [30], [31]. In neuroblastoma cells, the LIP is a function of total cellular iron, and an increase in cellular iron is accompanied by increased ferritin content to maintain the LIP within safe limits [32], [33]. Where might PrP intersect with this tightly orchestrated mechanism of iron uptake, transport, and storage? Three potential mechanisms are plausible: 1) modulation of uptake at the plasma membrane independently or by interacting with the Tf/TfR dependent pathway, 2) facilitation of iron transport to cytosolic ferritin across the endosomal membrane by promoting ferric iron release from Tf and/or its reduction for transfer through DMT1 [19], or 3) assistance in deposition into ferritin by oxidizing ferrous iron to the ferric form. It is unlikely that PrP facilitates export of iron from neuroblastoma cells based on our observations.

At the plasma membrane, PrP^C^ could take up iron directly from the extra-cellular milieu and deliver to an endosomal compartment as suggested for copper [34]. However, this seems unlikely for three reasons; 1) ^59^Fe-labeled PrP^C^ could not be detected in radiolabeled cells although labeled recombinant PrP is easily detected using the same procedure [17], 2) ^59^Fe-labeled recombinant PrP loses its label to Tf when added to cells, indicating lower affinity for iron relative to Tf (unpublished observations), and 3) intra-cellular LIP is high in cells expressing anchorless PrP^231stop^ despite low ferritin iron content, indicating efficient uptake of iron in the absence of cell surface PrP^C^. It remains plausible, though, that PrP^C^ modulates iron uptake by the Tf/TfR pathway at the plasma membrane or in an endosomal compartment [35].

It is also possible that extracellular iron induces the movement of PrP^C^ from detergent insoluble membrane domains where it normally resides to the proximity of TfR in a similar manner as in the presence of copper [34]. Here, it may enhance the binding of iron loaded Tf to its receptor, or stimulate the endocytosis of Tf/TfR complex by a direct or an indirect interaction. In this context, it is interesting to note that PrP^C^ undergoes endocytosis through clathrin coated pits after associating with a transmembrane protein through its N-terminal domain [36], suggesting that the reported co-localization of PrP^C^ with Tf and TfR within endosomes may reflect a functional association rather than co-residence due to a common mode of endocytosis [37]. Assuming this scenario, the increase in TfR levels by stimulation of PrP endocytosis by 3F4 and the differential effect of mutant PrP forms on ferritin iron content may be explained by a change in the rate of endocytosis, or altered interaction of normal and mutant PrP forms with Tf or TfR due to misfolding [35]–[37]. We have previously reported increased endocytosis and defective recycling of mutant PrP^102L^ in neuroblastoma cells [38], a fact that may account for increased ferritin iron in these cells. Though attractive, this model fails to explain decreased ferritin iron in the presence of significantly high LIP in cells expressing anchorless PrP^231stop^ and by cross-linking PrP at the plasma membrane, indicating a role downstream from the plasma membrane. The up-regulation of PrP^C^ at the transcriptional and translational level when cells are exposed to excess extra-cellular iron (supporting information) perhaps reflects its function as an iron regulatory protein, though a protective response to oxidative stress cannot be ruled out under these experimental conditions [39]. However, since all cell lines display similar differences in ^59^Fe-ferritin content when labeled with ^59^FeCl_3_ or purified ^59^Fe-Tf (unpublished observations), it is likely that PrP^C^ functions downstream of the iron uptake pathways specific for free and Tf bound iron, perhaps in an endosomal compartment.

Keeping the above facts in mind, it is plausible that PrP^C^ functions as a ferric reductase along with Steap3 to facilitate the transport of ferric iron released from Tf across the endosomal membrane to cytosolic ferritin. This assumption is supported by the fact that PrP^C^ functions as a copper transport protein by reducing copper (II) prior to transfer to copper (I) specific trafficking proteins within cells [34]. Such a function would explain the low ferritin iron content in cells expressing mutant PrP lacking the octapeptide region responsible for reducing copper (II) [34], the observed up-regulation of PrP^C^ in response to exogenous iron, increase in ferritin iron by increased expression of PrP^C^ and stimulation of PrP endocytosis, and co-localization of PrP^C^ and ferritin in cells exposed to excess iron [17]. However, decreased ferritin iron despite high LIP levels in cells expressing anchor-less PrP and the opposite scenario in PrP^102L^-cells suggests an additional role in iron transport between the LIP and cellular ferritin, a function that is hard to explain merely by the altered reductase activity of mutant proteins. Although we could not detect measurable ferroxidase activity of recombinant PrP, such a function of cell associated PrP^C^ would explain the facilitative effect of PrP^C^ on iron incorporation into ferritin. Further studies are required to resolve this question.

Despite obvious shortcomings in our data in explaining the mechanistic details of cellular iron modulation by PrP, this report clearly shows the effect of PrP and its mutants on iron uptake and transport. We demonstrate a state of mild iron overload mediated by PrP^C^, and mild iron deficiency or imbalance by pathogenic and non-pathogenic mutations of PrP. The positive role of PrP^C^ on cellular iron levels is further supported by a recent study where transgenic mice lacking PrP^C^ expression (PrP^−/−^) recover slowly from experimentally induced hemolytic anemia [40], indicating a functional role for PrP^C^ in iron uptake by hematopoietic cells. These findings take on a greater significance since prion disease affected human and animal brains show signs of iron imbalance [45], a potentially neurotoxic state due to the highly redox-active nature of iron. It is conceivable that dysfunction of PrP due to aggregation combined with the formation of redox-active PrP^Sc^ aggregates [17] induces brain iron imbalance, contributing to prion disease associated neurotoxicity. Future studies are required to define the precise biochemical pathway of iron modulation by PrP, and develop therapeutic strategies to prevent iron induced neuronal death in prion disorders.

## Materials and Methods

### Antibodies and chemicals

Monoclonal anti-PrP antibodies 3F4 and 8H4 were obtained from Signet (Dedham, MA) and Drs. Man-Sun Sy (Case Western Reserve University) and Pierluigi Gambetti (National Prion Surveillance Center, Case Western Reserve University) respectively. Antibody against human ferritin was purchased from Sigma (St. Louis, MO), anti-transferrin from GeneTex (San Antonio, TX), anti transferrin receptor from Zymed Laboratories Inc (Carlsbad, CA), and anti-Thy 1.1 from eBioscience (SanDiego, CA). Secondary antibodies tagged with HRP or fluorophores FITC and TRITC were obtained from Amersham Biosciences (England) and Southern Biotechnology Associates (Birmingham, AL) respectively. Ferrous ammonium sulfate, Ferene S, and all other chemicals were purchased from Sigma. All cell culture supplies were obtained from Invitrogen. ^59^FeCl_3_ was from Perkin-Elmer.

### Cell lines and culture conditions

Human neuroblastoma cells (M17) were obtained from J. Biedler (Memorial Sloan-Kattering Cancer Center, New York) and purchased from ATCC. M17 cells expressing PrP^C^, PrP^231stop^, PrP^Δ51–89^, PrP^Δ23–89^, and PrP^102L^ were generated and cultured as described in previous reports [41], [42]. For this study M17 cells from two different sources were transfected at least three separate times and bulk transfected cells were used to avoid cloning artifacts. Similarly transfected cells from two different investigators and cells cultured in DMEM supplemented with 10% FBS and Opti-MEM supplemented with different lots of FCS were also tried to avoid errors due to culture conditions.

### Radiolabeling with ^59^FeCl_3_

M17, PrP^C^, and mutant PrP^Δ51–89^, PrP^Δ23–89^, PrP^102L^, and PrP^231stop^ cells cultured overnight to 80% confluency were serum starved for 1 h and incubated with ^59^FeCl_3_-citrate complex (1 mM sodium citrate and 20–25 µCi of ^59^FeCl_3_ in serum free Opti-MEM; molar ratio of citrate to iron was maintained at 100∶1) for 4 h at 37°C in the incubator. At the end of the incubation cells were washed 3 times with ice cold PBS and lysed with native lysis buffer (0.14 M NaCl, 0.1 M HEPES, pH 7.4, 1.5% Triton X-100 and 1 mM PMSF). Aliquots of lysates were mixed with glycerol (to a final concentration of 5%) and traces of bromophenol blue, and equal amount of protein from each sample was resolved on 3–9% native gradient gel. For fractionation on SDS-PAGE, the same samples were mixed with 4× SDS-sample buffer, boiled for 10 min and resolved on SDS-PAGE followed by immunoblotting.

### Native gradient gel electrophoresis, autoradiography, immunoblotting and electroelution

Electrophoresis of lysates was performed using a Hoefer SE 600 vertical apparatus with a cooling system. Linear 3–20% (Fig. 1) or 3–9% (Fig. 3) gradient polyacrylamide gels were prepared as described by Vyoral et al. [21] with modifications. The gel mixture contained 0.375 M Tris, pH 6.8, 1.5% Triton X-100, and 1.18 mM ammonium persulfate. *N*,*N*,*N′*,*N′*-Tetramethylethylenediamine (TEMED) was added to a final concentration of 5.38 mM. Radiolabeled lysates mixed with glycerol were subjected to electrophoresis using electrode/running buffer (25 mM Tris, 192 mM glycine pH 8.3, and 1.5% Triton X-100) under constant current (100 mA) for 4 h at 4°C. Gels were either electroblotted or vacuum dried (BioRad) and exposed to X-ray film (Kodak BioMax XAR) fitted with intensifying screens. For Western Blotting, gels were washed thoroughly with electrode buffer without Triton X-100 for 2 h (each wash of 200 ml, 10 min) on a slowly rocking platform to remove Triton. The gel was electroblotted to a PVDF membrane using BioRad semi-dry electroblotting system with anode buffer (25 mM Tris, pH 10.4) and cathode buffer (25 mM Tris, 39 mM glycine, pH 9.2) at 25 V for 90 min. Membranes were further processed for immunodetection as described below. To confirm the identity of iron labeled proteins, iron bands were excised from native gels and proteins were electro-eluted using Biorad electro-eluter at 60 mA for 4 h. Eluted proteins were concentrated by methanol precipitation and analyzed by SDS-PAGE.

### SDS-PAGE and Western blotting

Cells cultured under different conditions were fractionated by SDS-PAGE and immunoblotted as described previously [41], [42]. The following antibody dilutions were used: 8H4 (1∶3000), 3F4 (1∶5000), ferritin (1∶1000), Tf (1∶6000), TfR (1∶3000), actin (1∶7500), secondary antibodies conjugated with horseradish peroxidase (1∶6000). Immunoreactive bands were visualized by ECL (Amersham Biosciences Inc.).

### Measurement of intracellular calcein-chelatable iron

Cellular labile iron pool (LIP) was assayed as described by Tenopoulou et al. [43] using the iron sensitive fluorescent dye calcein. When incubated with cells as a lipophilic calcein-AM-ester (molecular probes), it enters the cells and is cleaved by cellular esterases to release calcein that binds iron and is quenched by this reaction. Upon addition of the cell permeable iron chelator salicylaldehyde isonicotinoyhydrazone (SIH), iron is released from calcein that regains its fluorescence (recorded at λ_ex_ 488 nm and λ_em_ 518 nm). Briefly, 5×10^5^ M17 cells or cell lines expressing PrP^C^ and mutant PrP forms plated in 35 mm Petri dishes were washed with PBS containing 1 mg/ml BSA and 20 mM Hepes, pH 7.3 and incubated with 0.25 µM calcein-AM for 20 min at 37°C in same buffer. After calcein loading, cells were trypsinized, washed and re-suspended in 1.0 ml of the above buffer without calcein-AM and placed in a 24 well micro-plate in a thermostatically controlled (37°C) fluorescence plate reader (Microtek). The fluorescence was monitored at λ_ex_ 488 nm and λ_em_ 518 nm. Iron-induced quenching of calcein was reduced by the addition of 20 µM SIH. Cell number and viability was checked by Trypan Blue dye exclusion and results were expressed as ΔF/10^6^ cells.

### Detection of iron with Ferene S

Cell lines cultured overnight in complete medium or in the presence of 0.1 mM ferric ammonium citrate (FAC) were washed with PBS supplemented with EDTA to chelate surface bound iron and pelleted. The pellet was dissolved in 50 µl of acetic acid and equal amount of protein (50 µg) was spotted on a PVDF membrane and immersed in a freshly prepared solution of Ferene S (0.75 mM 3-[2-pyridyl]-5, 6-bis(2-[-furyl sulfonic acid]-2, 4-triazine, 2% (v/v) acetic acid, 0.1% thioglycolic acid) (24) for 30 minutes at 37°C. Ferene reacts with iron in the presence of acetic acid and thioglycolic acid to form a dark blue complex. Stained membranes were de-stained with 2% acetic acid and scanned.

### Stimulation of endocytosis with 3F4 antibody

M17 and PrP^C^ cells were cultured in DMEM supplemented with 5% FBS and 1% PSF at 37°C in a humidified atmosphere in absence or presence of 1 µg/ml of 3F4 for 5 days [26], [27]. Medium containing 3F4 was replaced every 2^nd^ day and care was taken to make sure that the cells did not achieve confluency. On the 5^th^ day, cells were washed and incubated with serum free DMEM for 1 h, followed by radiolabeling with ^59^FeCl_3_-citrate complex in DMEM for 4 h as above. In a separate experimental paradigm, N2a, M17 and PrP^C^ cells were radiolabeled as above in the presence of 12 µg/ml of 3F4 or Thy-1 4 h. After labeling, cells were washed, lysed in native lysis buffer, and analyzed as above.

### Immunostaining and fluorescence microscopy

Cell lines subjected to different experimental conditions were processed for immunostaining as described in a previous report [41].

### Estimation of iron export from cells

Cell lines expressing different PrP forms were radiolabeled with ^59^FeCl_3_-citrate complex as above. Cell surface bound iron was chelated with 3 washes of PBS supplemented with DFO (100 µM) and the cells were chased in complete medium for different time periods. A 50 µl aliquot of the medium was retrieved at each time point and counted in a γ-counter. After 16 h, cells were lysed and cell associated iron was measured in a gamma counter.

### Estimation of ferroxidase activity of recombinant PrP

Ferroxidase activity of PrP was measured by the published colorimetric method using 3-(2-pyridyl)-5,6-bis(2-[5furylsulfonic acid])-1,2,4-triazine that forms a colored Fe^2+^ complex with ferrous iron (44) with the following modifications: Reagent A: 0,45 mol/l sodium acetate, pH 5.8, reagent B: 130 mmol/l thiourea, 367 µM/l Fe(NH_4_)(SO_4_)_2_×6H_2_O, reagent C (chromogen): 18 mmol/l 3-(2-pyridyl)-5,6-bis(2-[5-furylsulfonic acid])-1,2,4-triazine in 0.01 M Tris pH 7.0. Each sample contained either 1 µl of water or 1 µl of 300 µM CuSO_4_, 6 µL of the sample (undiluted human plasma, human serum albumin 70 g/l (Sigma A1653-5G) in PBS or recombinant prion protein (0.6 µg/ml) and 820 µl of reagent A. Multichannel pipette (Finnpipette) was used for the rapid addition of the reagent B (substrate) to minimize the time difference in sample processing. Sample quadruplicates were incubated at 37°C for 4 min. Unoxidized Fe^2+^ was reacted with 60 µl of chromogen solution (reagent C) and absorbance was measured at 600 nm with Smart Spec Plus (BioRad) spectrophotometer. Copper was added to provide two copper ions per PrP molecule, and was also added to human albumin and plasma samples. The amount of PrP protein in PrP-containing samples (3.6 µg/sample) roughly corresponds to a known amount of ceruloplasmin in 6 µl of undiluted human plasma. As a control, purified 99% human serum albumin was used (70 g/l in PBS) to mimic the total protein concentration in plasma. As a blank samples were supplemented with 6 µl of de-ionized water instead of albumin solution, plasma or recombinant PrP solution.

### RNA Isolation and Northern blotting

M17 and WT cells cultured in the absence or presence of 0.1 mM FAC for 24 h were washed with cold PBS, trypsinized, and collected in 1.5 ml eppendorf tubes. Total RNA was isolated by using SV total RNA isolation kit (Promega, Madison, WI) and quantified. 15 µg of total RNA was fractionated on 0.8% formaldehyde agarose gel followed by blotting to positively charged Nylon membranes (Roche diagnostics). Membranes were hybridized with DIG-labeled PrP or β-actin probes and binding was detected by the CSPD reagent.

### Statistical analysis

Data are presented as the mean±SEM values. Statistical evaluation of the data was performed by using Students t-test (unpaired).

## Supporting Information

Figure S1 (A) Apotransferrin (Sigma) was radiolabeled with 59FeCl3-citrate complex and resolved on a native gel as in Fig. 2. Tf migrates as three distinct bands representing different conformational forms. (B) Autoradiographed gel from Figure 2 was re-hydrated and stained with silver to ensure equal loading of proteins (Beta-actin does not resolve on this native gel).
(7.48 MB TIF)

Figure S2 Lysates of PrPC cells labeled with 59FeCl3-citrate complex were resolved on native gel as in Fig. 1 and exposed to an X-ray film to visualize iron labeled bands (panel A). Marked areas were excised from the wet gel, proteins were electro-eluted, and resolved by SDS-PAGE followed by sequential immunoblotting with antibodies specific to PrP, TfR, ferritin, and Tf (panel B). Finally, the membrane was stained with silver to visualize all proteins (panel B). Band 1 that includes proteins in the loading well reacts strongly for PrP and TfR. Band 3 reacts specifically for ferritin, while band 5 represents Tf. No detectable proteins are present in band 4. Silver staining shows 4 prominent proteins in bands 1–3, the identity of which is currently unknown.
(10.07 MB TIF)

Figure S3 (A) Lysates of M17 and PrPC-cells treated as in Figure 5 were fractionated by SDS-PAGE and transferred proteins were probed for PrP, ferritin, TfR, and β-actin (lanes 1–4). (B) FAC exposed M17 and PrPC-cells show up-regulation of PrP mRNA compared to untreated controls (lanes 1–4).
(2.03 MB TIF)

Figure S4 M17-cells exposed to buffer, 3F4, and anti-Thy-1 antibody were radiolabeled with 59FeCl3-citrate complex and lysates were resolved on native gel followed by autoradiography (lanes 1–3). Equal aliquots of the same samples were resolved by SDS-PAGE followed by immunoblotting for β-actin to ensure equal loading of protein (lanes 1–3).
(1.00 MB TIF)
