# Correction: Prion Protein Modulates Cellular Iron Uptake: A Novel Function with Implications for Prion Disease Pathogenesis

**DOI:** 10.1371/annotation/194f4e44-20f0-48eb-bbe9-14e21d18909b

**Published:** 2009-02-19

**Authors:** Ajay Singh, Maradumane L. Mohan, Alfred Orina Isaac, Xiu Luo, Jiri Petrak, Daniel Vyoral, Neena Singh

The following information was omitted from the funding statement: This study was supported by NIH grants NS39089 and NS35962 to NS and MSMT CR grants LC06044 and 002162080 to JP and DV.

