# Supplementary figures and images for: Prion Protein Modulates Cellular Iron Uptake: A Novel Function with Implications for Prion Disease Pathogenesis
Source: PLoS One. 2009 Feb 12;4(2):e4468. doi: 10.1371/journal.pone.0004468 (PMC2637434; doi:10.1371/journal.pone.0004468)

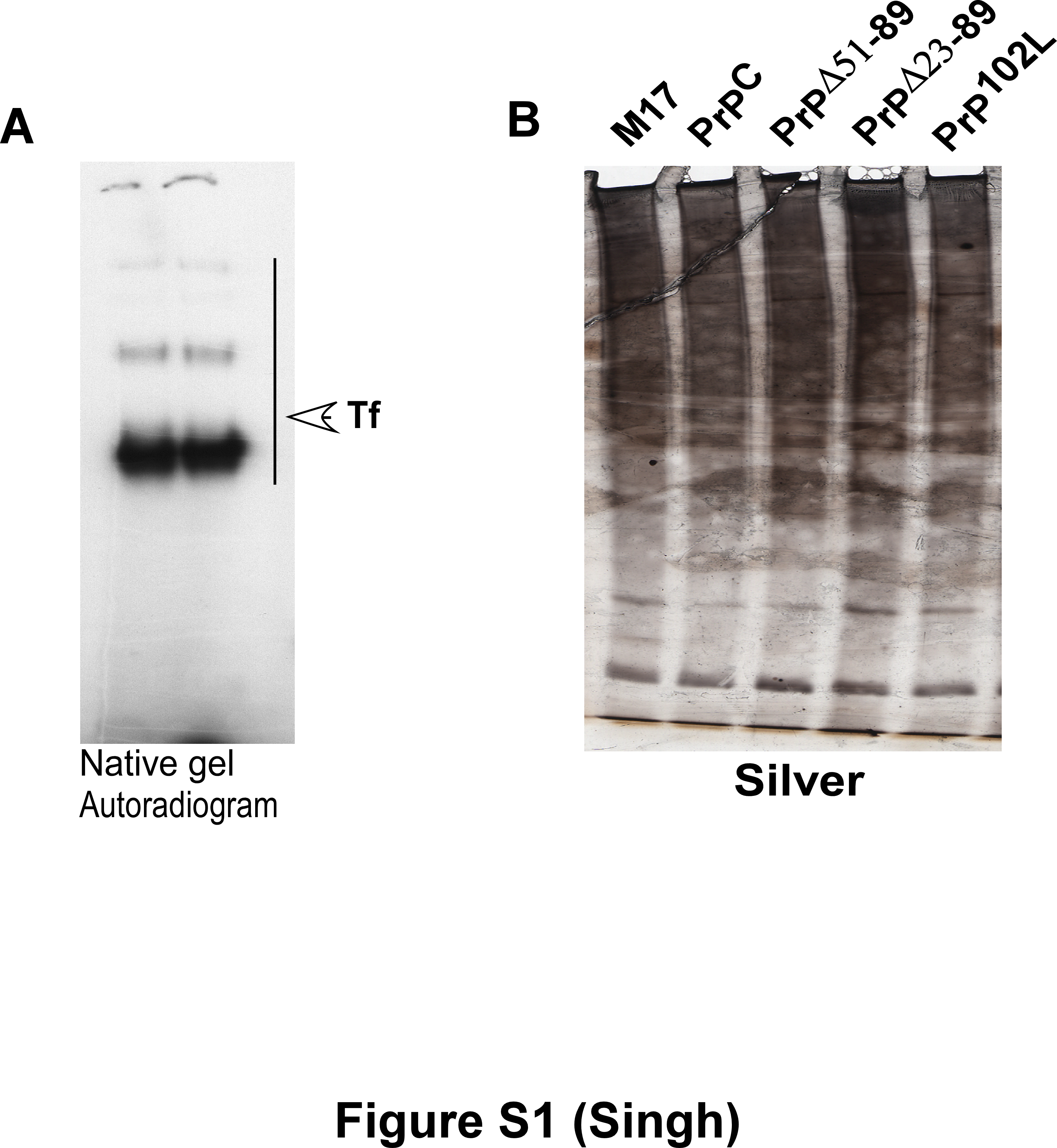

Supplement: Figure S1 — (A) Apotransferrin (Sigma) was radiolabeled with 59FeCl3-citrate complex and resolved on a native gel as in Fig. 2. Tf migrates as three distinct bands representing different conformational forms. (B) Autoradiographed gel from Figure 2 was re-hydrated and stained with silver to ensure equal loading of proteins (Beta-actin does not resolve on this native gel). (7.48 MB TIF) [file pone.0004468.s001.tif]

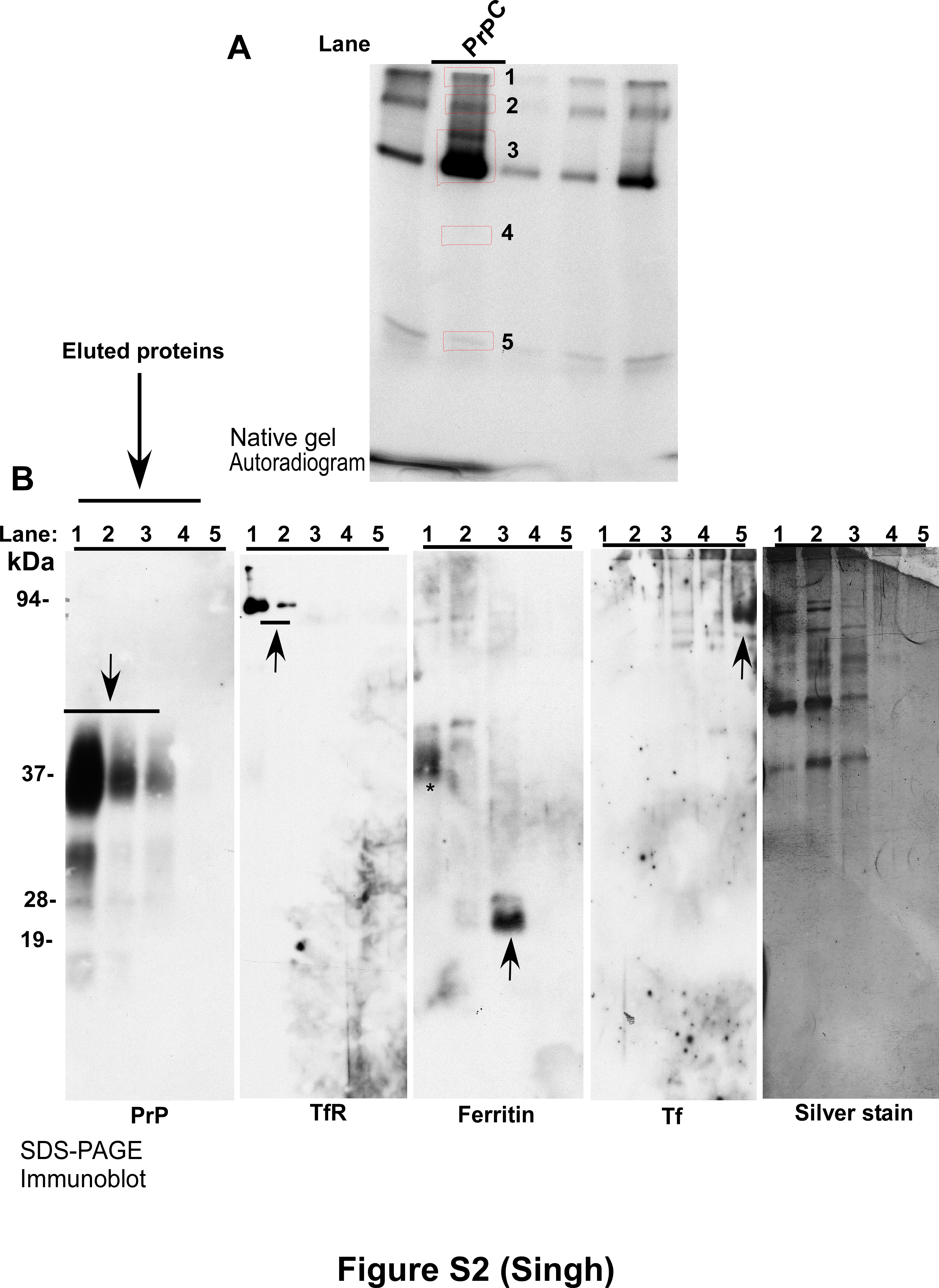

Supplement: Figure S2 — Lysates of PrPC cells labeled with 59FeCl3-citrate complex were resolved on native gel as in Fig. 1 and exposed to an X-ray film to visualize iron labeled bands (panel A). Marked areas were excised from the wet gel, proteins were electro-eluted, and resolved by SDS-PAGE followed by sequential immunoblotting with antibodies specific to PrP, TfR, ferritin, and Tf (panel B). Finally, the membrane was stained with silver to visualize all proteins (panel B). Band 1 that includes proteins in the loading well reacts strongly for PrP and TfR. Band 3 reacts specifically for ferritin, while band 5 represents Tf. No detectable proteins are present in band 4. Silver staining shows 4 prominent proteins in bands 1–3, the identity of which is currently unknown. (10.07 MB TIF) [file pone.0004468.s002.tif]

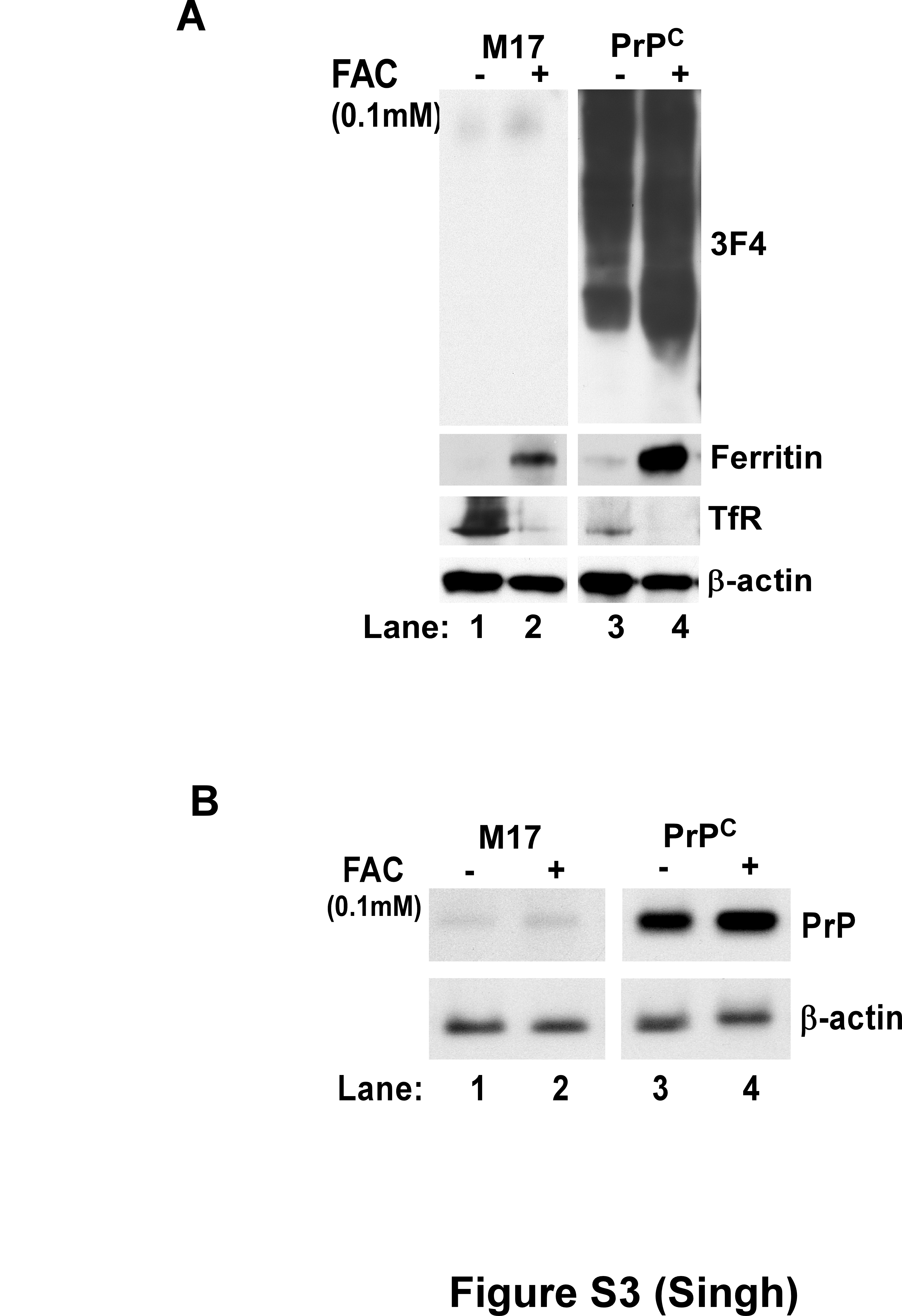

Supplement: Figure S3 — (A) Lysates of M17 and PrPC-cells treated as in Figure 5 were fractionated by SDS-PAGE and transferred proteins were probed for PrP, ferritin, TfR, and β-actin (lanes 1–4). (B) FAC exposed M17 and PrPC-cells show up-regulation of PrP mRNA compared to untreated controls (lanes 1–4). (2.03 MB TIF) [file pone.0004468.s003.tif]

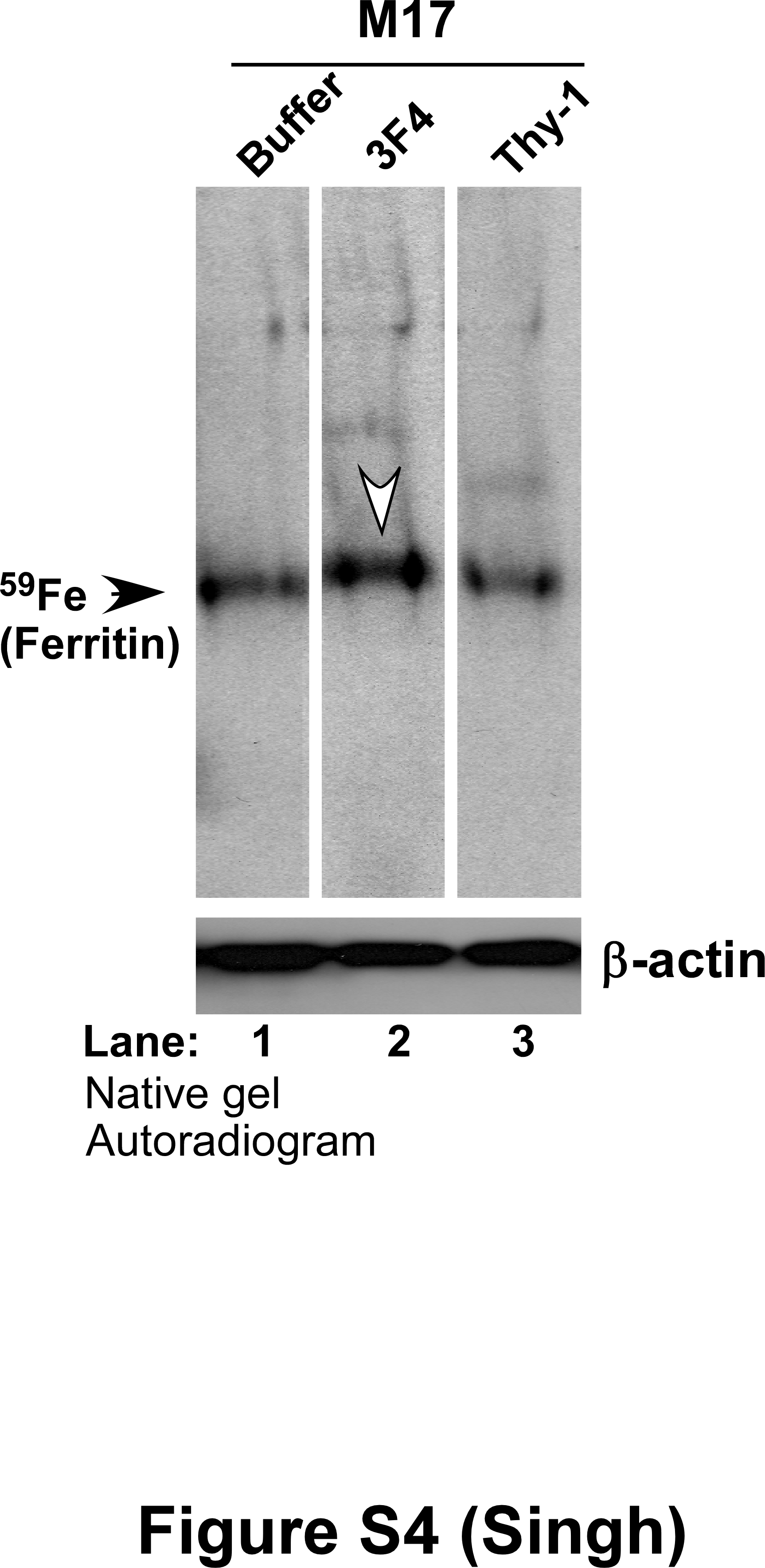

Supplement: Figure S4 — M17-cells exposed to buffer, 3F4, and anti-Thy-1 antibody were radiolabeled with 59FeCl3-citrate complex and lysates were resolved on native gel followed by autoradiography (lanes 1–3). Equal aliquots of the same samples were resolved by SDS-PAGE followed by immunoblotting for β-actin to ensure equal loading of protein (lanes 1–3). (1.00 MB TIF) [file pone.0004468.s004.tif]
